# Book Reviews

**Published:** 1822-03

**Authors:** 


### CRITICAL ANALYSIS

OF

### ENGLISH AND FOREIGN LITERATURE

RELATIVE TO THE VARIOUS BRANCHES OF

### Medical Science.

Quæ laudanda forent, et quæ culpanda, vicissim

Illa, prius, cretâ; mox hæc, carbone, *notamus.*—PERSIUS.

### DIVISION I.

### ENGLISH.

ART. I.—*The Principles of Medicine, on the Plan of the Beconian* [Baconian] *Philosophy. Volume First: on Febrile and Inflammatory Diseases.* By R. D. HAMILTON. 8vo. pp. 295. T. and G. Underwood, London. 1821.

SOME authors are unlucky in the choice of their subjects,—others in that of their style and turns of expression: Mr. HAMILTON, on the contrary, seems to have been particularly unfortunate in the selection of his printer. If the work before us be a fair specimen of the state of the typographic art in the northern metropolis, the sooner people there set about improving themselves the better. In the title-page we have *Beeonian* for BACONIAN; and, in the first page of the introduction, we meet with *erdulity* for credulity: *eminated* for emanated; *inconsistancies* for inconsistencies; *idiopothic* for idiopathic, &c. &c. But it is not so much with Mr. Hamilton's printer, as with Mr. Hamilton himself, that we feel disposed to quarrel. We doubt whether a work, professedly written with higher pretensions, and with openly-declared claims to a greater share of the public attention, than works of this kind are, in general, given to the world, has ever been found so miserably, so completely, to disappoint public expectation as the present one has done, and will do still more, if more generally read. Mr. Hamilton has placed his book under the protecting influence of an illustrious name. He has invoked the genius of BACON to assist him in his performance; and, in order to guide the medical profession, *ipse duce*, towards a better mode of judging diseases, he has suddenly turned the tables upon us, and has declared that we ought to judge by the effect, and not by the cause.

If, with all this appearance of novelty in medical logic, our readers should expect to find much that is new in the manner of considering, describing, or treating diseases, they will be greatly disappointed. The author's claim to originality is strictly limited to the *title* of his work, and to a few odd principles started in the introduction. The diseases themselves are described much in the usual manner of all other writers on practical medicine; namely, by stating their history, symptoms, causes, and treatment. When we add that, in speaking on the three first points, our author is deficient, rather than copious and exact, and that he has advanced nothing, of any consequence, which may be fairly considered as new in his treatment of diseases—we think we have fairly pencilled the outline-sketch of Mr. Hamilton's performance.

The work contains the history of various diseases, so happily jumbled together, with an entire disregard to classification, that we find hydrophobia, for instance, closely wedged-in between enteritis and hepatitis.

The author's ideas, however, both with regard to the mode of studying medicine, in ancient as well as modern times, and to the etiology of diseases, are best collected from his introduction. This elaborate essay presents us, also, with a fair specimen of his style and rhetorical powers. It will, therefore, engage the best part of our consideration. It will go hard with us, indeed, if we do not prove, ere we have done with this very introduction, that the author has written his own sentence, and that of his book, when he stated that:

"While a people are only bordering on learning and refinement, we generally find their fancy strong, and their reason weak; their writings always hypothetical, and crowded with flights of imagination, to clothe which with appropriate language, they naturally select the most sonorous expressions, till, deluded by the harmonious junction of periods, they substitute sound for sense, and drown their arguments in a torrent of words."

Mr. Hamilton contends that the medical world was bewildered, from the time of Galen, until the appearance of Dr. Brown, who, "in his Elements of Medicine, was the first that attempted to turn the goal, and give medical inquiries a new direction, in opposition to Cullen, who seems to have been the last of the systematic visionaries."

"Reciprocally inflamed," observes Mr. Hamilton, "by the specious vagaries of the day,—at the same time influenced by the natural propensity to imitation, they seem to have considered the monitor reason as a delusive criterion, and vied one with another only in producing incredible fictions. Hence spring the unmeaning terms *error loci*, *morbid lentor*, and *spasm*, besides ten thousand mouthfuls of nonsense, that have retired to the shades; there to rot amidst the lumber of antiquity, in company with other popular errors that formerly misled mankind."

Having descanted on the errors of those that are gone, the author prepares to develop his own system; but, in doing this, he "is willing to forego the custom of overturning antecedent fabrics;" and for this very sensible reason, that:

"It is not the voice of one, however loudly exerted, but a number of intelligent and experienced practitioners, superior to prejudice, and deaf to the despotic roar of opinion, who are the only jury competent to determine the truth, or fallacy, of medical propositions."

It is shrewdly observed by the author, that the animal frame ought to be considered, as it really appears to be, a piece of complicated machinery, kept in motion, not by the powers and qualities of a vital principle of which we know nothing, but by food, drink, air, and heat. "These stimulating agents," says Mr. Hamilton, "are known to every one, and familiar to the meanest capacity." There is no questioning the Baconian simplicity of this preliminary proposition.

What follows is equally simple:

"From the above doctrine we may rationally premise, that great physical strength in the various organs, the moderate and equitable application of all the stimulating agents, will generally procure health and ensure longevity. But the animal machine, when stimulated into violent action, by whatever power, like every other piece of machinery, when driven with extraordinary velocity, is soon worn out, deranged, or broken. Should no constitutional derangement take place, some one or other of the organs, weaker than the rest, fails to perform its proper functions; or if, by a more accurate balance, the whole organs maintain an uninterrupted harmony, old age advances before its proper time, and the whole system, exhausted, like an automaton that has spent its cord, stands still for want of impulse. Hence, the almost *unsufferable* stimulus of heat, the pungent liquors, and burning spices, so universally used as table necessaries, are apparent causes, that render the beautiful plains of the East deleterious to health, and the tropical climates the early grave of Europeans. The negro, almost melting under the *perpendicular* rays of a *vertical* sun, and enjoying the luxuries of the torrid zone, is hurried through all the stages of life with amazing rapidity, and sinks of inanition at an early age. The same observation is applicable to the vegetable kingdom: our forest-pines, when planted on the chill mountains of Norway, are longer in reaching the zenith of maturity, than those which adorn the fertile plains of America: but the former are firmer in their texture, and retain their verdure when the latter are rotten with age. In like manner, the shivering inhabitant of the North, whose blood scarcely creeps through his veins, wrapped in fur, seeking a scanty subsistence on his frozen shores, often survives his three-score years and ten. We have a still more familiar example exhibited in the infatuated drunkard, whose animal frame is continually stimulated into preternatural action by habitual intoxication. He is soon emaciated to a skeleton, —his trembling limbs announce the decay of nature, and his moribund looks proclaim the *lease* of existence drawing to a close: whilst the abstemious peasant preserves an erect figure and a firm step, under the hoary hair and venerable aspect of three-score. But, with every advantage, we are but the ephemeral beings of a day; for, after a few transient years, the animal frame becomes callous, and incapable of preserving its momentum, though excited by the most powerful stimulating agents,—the bones begin to grow brittle,—the hairs to fall from the head,—the teeth to drop from their sockets,—and the whole body continues gradually to waste, until some trifling disease overwhelms the remaining strength, and death concludes the scene."

The above is a picture of the animal frame in a state of health and "*unmorbid* decay." Its operations in a state of disease are next considered.

When we come to the bed-side of the patient, (we repeat, nearly verbatim, the author's own words,) our first duty is to examine—what are the deviations from health; where do they affect, as the primary seat of disease; and, lastly, understand what the *morbid action* really is. Now, as no two diseased actions of an opposite nature, observes Mr. Hamilton, can exist in the same constitution at the same time, "the stronger necessarily expelling the weaker, as heat overcomes cold, water thirst, or light darkness," it follows that, if we can induce an opposite action, sufficiently powerful to expel the diseased one from the system, we shall be certain of methodically and rationally curing disease. The disease itself Mr. Hamilton distinguishes by the appellation of the *morbid action*, for the sake of perspicuity; the opposite, or curing action, by the appellation of *counteracting action.* So here have we a Baconian philosopher, who wishes to take nothing for granted that he cannot prove,—who hates metaphysics in medicine, because they give to "airy nothing a local habitation and a name,—assuming the existence of two abstract entities, and those entities actions, one of which is to expel the other! One *preternatural* action to expel another action!" *Proh pudor!* "But I hope," remarks Mr. Hamilton, "that I am expressing myself perspicuously."—Very much so, indeed! Take these as examples:

"The morbid action of colic is merely a remora in the bowels, which is removed by the counteracting action of purging. In common catarrh, the morbid action is plainly an obstruction of the cutaneous perspiration, and is readily relieved by the counteracting action of diaphoresis; after which, the defluction from the lungs, and the running at the nose, cease."

Now, in the name of good sense, what is there in all this, supposing it even to be true, that has not been stated a hundred times before, by the very "absurd ancestors" whom our author has sweepingly stigmatized? Has not every one of them asserted, and repeated, that the best cure for constipated bowels is purging; and the surest mode of obviating the bad effects of checked perspiration, is to induce *diaphoresis?*

But, let us proceed in perfect good-humour.—When the *morbid action* is really ascertained, and the species of *counteracting action*, "which will most effectually destroy it," is determined upon, we have yet one great point to take into consideration. Is the system capable of bearing the introduction of a *counteracting action* stronger than the *morbid* one? Having held council upon this, and satisfied ourselves that the two actions may be allowed to come to close quarters without destroying the patient, hear what our *military* author has to suggest in this matter:

"We must, on no account, procrastinate the cure until the disease acquire strength. By employing our means alternately in succession, we must attack the disease at every point where it is vulnerable, concentrating all our forces at once, like a grand military movement, employing as many remedies as can be brought to bear upon the disorder at one time, and sweep it from the system by a synchronous charge."

If the patient possess sufficient strength to bear this engagement, the author assures us that *he must* be cured, and that the thing "is as plain as the chemical fact, that the stronger affinity will disengage the weaker; or the one in mechanics, that the greater weight will raise the lesser, when hung on the opposite ends of a steelyard."

But we fear we have been trifling too long with our readers, who must by this time perceive of what stuff our author's cranial brain is made. We are not given to satirical or humorous writing; but, surely, no editor of a critical Journal has ever had a better opportunity afforded to him of exercising both these qualities, than on the present occasion.

As for the style and language in which Mr. Hamilton's doctrines are brought forward, our readers must have had sufficient opportunities of judging of both, from the numerous extracts we have thought it necessary to adduce. A few more of these quotations will complete the picture.—MILTON is said to have written in a *romantic* style on the loss of Paradise. Some authors are *inflamed* by specious *vagaries*. Lines (we suppose he means parallel lines,) in mathematics, seem for ever approaching, but never coalesce. Spasm, morbid lentor, and error loci, with other equally-corporeal beings, such as "ten thousand mouthfuls of nonsense," (page 2,) are condemned to the shades, there to *rot* amidst the *lumber* of antiquity." Besides these specimens of eloquence and taste, we have many examples of sound expressions and purity of language, such as "acumen of exactness;"—"a philosopher wading, beyond his depth, in a wilderness of enigmas and a chaos of ideas;"—"the *perpendicular* rays of a *vertical* sun;"—"the *lease* of existence drawing to a close;"—"the current of the tide stopped by the pressure of a boy's hand;" &c. &c.

We now proceed to inquire into the claims of our Baconian philosopher to our admiration and thanks for any improvement he may have suggested, in the practical part of our profession.

Intermitting fever is the first subject treated of in this book. The description of it is thus given by the author:

"The paroxysms of an intermittent consist of a cold, a hot, and a sweating stage; which, for the sake of perspicuity, are generally described separately in their genuine order of succession: beginning with the cold stage, in which the pulse is weaker and slower than in a healthy state, frequently irregular, and sometimes almost imperceptible. From this low pulsation proceeds universal languor, which is felt in the whole system, through all the gradations of uneasy weariness, restlessness, yawning, stretching, numbness, stupor; and even the senses of sight and hearing are sometimes lost. At the extremities, where the circulation is weakest, the skin first begins to feel cold, and soon appears blue and shrivelled, particularly the toes, fingers, and lips; then gradually the whole skin becomes rough, like the skin of a fowl, or that of a person suddenly exposed to intense cold. The patient complains of nausea and sickness at stomach, and evidently labours under great dejection of spirits; a violent shivering immediately takes place; the eyes lose their lustre, and communicate a dull, melancholy stare; the teeth chatter, the patient draws himself together, and speaks and breathes with difficulty. Sometimes his voice is suppressed by anxious and deep sighing, and is continually accompanied with a peculiar sound, not unlike the accents of a person whose voice is broken with age. It was this pale shrinking of the lips and extremities that gave rise to the Cullenean spasm, which, like other absurd and useless theories, has had its day."

The intermediate stage of quivering between cold and heat is mentioned; and the increase of the latter, with its accompanying train of symptoms, is thus sketched:

"At this period, blood not unfrequently bursts from the eyes, nose, mouth, and other cavities of the body. The thirst, that was in some degree present from the beginning of the cold stage, now becomes unquenchable; the tongue brown and tremulous, dry and chipped; the urine high-coloured, but without sediment. The ideas flit through the mind with amazing rapidity, till, at last, getting bewildered, the senses themselves are shattered into delirium; and, in the headlong confusion, false hearing and false vision supervene. In the midst of this general anarchy, the sweat bursts forth, and a relaxation of all the excretories, accompanied with a loose stool, terminates this stage. The sweating, in general, makes its first appearance, like a dew, about the forehead and breast; then gradually extends over the whole surface of the body. The pulse now becomes soft and slower, the breathing free; the urine deposits a sediment like brick-dust; the patient falls asleep, and awakes much relieved. Still, however, he feels sore and greatly fatigued, has a sickly look, and a considerable degree of low fever remaining, which continues till the next paroxysm."

Mr. Hamilton contends that the definitions of quotidian, tertian, and quartan, so carefully preserved by Cullen in his Nosology, are useless distinctions,—the remains of a technical phraseology, which authors have not been able to lay entirely aside. In the course of his practice, our author found it impossible to trace any systematic regularity,—and was for ever calling this a quotidian, and that a tertian, confidently predicting the hour of the next paroxysm,—and was as regularly disappointed and astonished to find the revolutions for ever varying in the length of their duration. With regard to the diagnosis and prognosis in this disease, Mr. Hamilton has laid down the following laconic apophthegm:—"*Gather the first from the symptoms, the latter from their violence.*"

There is nothing satisfactory or new in the chapter of *causes* of intermitting fever; and, under the head of treatment, we looked in vain for a practical illustration of the author's doctrines of morbid action and counteracting action. What the *morbid action* of an intermitting fever is, has long been, and still is, the subject of much controversy, says Mr. Hamilton. Hence comes it, we suppose, that he has not specified the counteracting action which is to expel the former. The mode, therefore, of treating intermitting fever, laid down by the author, reduces itself to very much like what every other author has recommended, without the assistance of the great Lord Chancellor; so that, in this instance at least, the Baconian philosophy has yielded no assistance in improving medicine. There is, indeed, one point of Mr. Hamilton's practice, which may really prove a *counteracting* action in cases of intermitting fever; but whether of that kind to which the patient will be able to resist or not, we pretend not to say: we mean bleeding when the hot stage commences!

All intermittent fevers being dispatched under *one* head, we next proceed to the consideration of continued fevers; in treating of which, the author's description is, in a like manner, confined to one species,—namely *typhus.* "Synocha of the older physicians, is a fanciful disorder." "The yellow-fever of the West Indies, and the plague of Smyrna, are nothing more than typhus, aggravated by climate and other circumstances."

"To give a terse and luminous account of all the symptoms of typhus, is one of the most difficult subjects of description to be found in all the range of medicine; because it exhibits such an endless variety of symptoms, that authors have only the choice of being short and imperfect, or long and confused. It would, therefore, require the glowing thoughts and colouring expressions of a Shakespeare, a Byron, or a Scott, to do it justice."

The author then attempts the task, dipping his brush in the dark heavy tints of Carravaggio and Salvator Rosa. Alarming as all the symptoms are which he describes, they are not exaggerated. Mr. Hamilton says, Quæque ego miserrima vidi; "yet the very worst of them terminated favourably."

Of the causes of typhus, none are positive, except *contagion.* Wherever this acts, typhus is produced; but how contagion *acts*, is a mystery; so that, even in this second and formidable class of diseases, since the *morbid action* is unknown, the *counteracting* action, also, must be a secret.

Were we on any other subject of less importance, we should be very much disposed to laugh heartily at the author's good-natured, serious, sedate, unhesitating manner, of uttering the greatest absurdities that have ever been honoured with printer's ink. Contagion, says he, is probably an aëriform fluid. This probability is next changed into a physical certainty; for we are told, at page 50, that *contagion*, like carbonic acid, is heavier than atmospheric air. Yet the stories of fomites are stigmatized with the epithet of idle; and a *cutting* smile at the doctrine of contagion being carried from one house to another in clothes, or from one country to another in a bale of goods, is the precursor of the following exhortation:—Let "England burn her yellow flags, and do the country service, by converting her quarantine cutters into oyster smacks!"

Inflammation.—Under this, by far the largest division of the volume before us, Mr. Hamilton has arranged, and treated of, 1. Phlegmon; 2. Venereal Disease; 3. Ophthalmia; 4. Phrenitis; 5. Pneumonia; 6. Influenza; 7. Phthisis Pulmonalis; 8. Enteritis; 9. Hydrophobia; 10. Hepatitis; 11. Dysentery; 12. Cynanche Tonsillaris; 13. Cynanche Maligna; 14. Cynanche Trachealis; 15. Erysipelas; 16. Rheumatism; and 17. Gout.

We cannot be expected to enter into an examination of any one of the various subjects enumerated by the author. Much may be collected of what we omit, from what we have already stated; and, when we assure the readers that, neither as a work containing original thoughts of value, nor as one of reference for the correct exposition of well-known facts, the volume before us can be recommended to their attention, we trust we shall not be taxed of unjust severity or wilful cecity to the merits of Mr. Hamilton's production. We do not wish to prevent the public from reading his book; nay, we strongly urge them to consult it,—to read it, and then to judge.

We will only instance the author's definition of dysentery, as a fair specimen of his Baconian manner of conveying to his readers the ideas he entertains of the nature of that disease, in order that they may see how far his descriptions tally with those with which we are best and most familiarly acquainted.

"*Dysentery.*—This disease is a retention of indurated feces, sticking in the alimentary canal, and occasioning an inflammation of the intestines, accompanied by typhous fever. The feces that are retained in the cells of the colon form into hard masses, resembling small chesnuts, and are called scybale. These scybale, as they lie irregularly, only produce a partial obstruction in the alimentary canal; for, if the obstruction were complete, the disease would be colic, not dysentery. In consequence of this partial obstruction, the wind is frequently pent up among the scybale for a few minutes, when the inflamed intestine immediately becomes painfully distended, and continues so until the wind finds its way, which it does with a rumbling noise, occasioning a temporary relief, until it again meets with a new obstruction. This is the occasion of those violent gripings in the bowels, so distressing in dysentery."

Does the idea of the complaint in question, suggested by the above definition, answer to that contained in the few following words of Cullen:—"*Dysenteria.* Pyrexia contagiosa, dejectiones frequentes, mucosæ, vel sanguinolentæ, retentis plerumque fœcibus alvinis; tormina, tenesmus." Or does it correspond with the brief, but expressive, definition of Sauvages,—"Frequens torminosa, sæpe tenesmoda fœcum cruertarumque per inferiora dejectio?" or to the more explicit definition of its characters contained in the following lines:—"La dysenterie se reconnait aux caractères suivans: tranchées, ardeur dans le gros intestin, epreintes, tenesme, besoin frequent, irresistible, et par fois continuel, d'aller à la selle accompagné de violens efforts; evacuation, en tres petite quantité, d'une matiere muqueuse ou puriforme, souvent melée de sang; d'autres fois purement sanguinolente. Odeur specifique, extremement fétide, et nauseabonde des dejections. Fièvre dans la plupart des cas." Who is there that does not immediately recognize the disease intended to be pourtrayed in the above definitions?

The third division of Mr. Hamilton's book is on exanthematous diseases. Small-pox—Vaccination and secondary Small-pox—Measles—Scarlatina, are the complaints treated of in this part of the work. There are some few observations on Hemorrhagy; and the first volume concludes with—

"Such are the leading opinions which I have formed, relative to the laws of the human constitution, and the method of curing diseases. How far they are correct, or to what degree they are susceptible of improvement, future observations and experience will determine. The aim of this work has been to discover the principles of the science, and to fix the practice of medicine on a rational basis, where inferences and facts may follow one another, as clearly as in trigonometry."

Need we say, that Mr. Hamilton, in our humble opinion, has missed his aim; and that, with talents of no despicable nature, he has produced a book, which is not likely to benefit the profession, raise the author's name, or uphold the reputation of the "northern city!"

---

ART. II.—*A Series of Lectures on the most approved Principles and Practice of Modern Surgery; principally derived from the Lectures delivered by* ASTLEY COOPER, Esq. F.R.S. &c. &c. &c. *at the united Hospitals of Guy and St. Thomas; and in which will be found some of the Opinions of the most celebrated Surgeons, from the time of Hunter to the present moment. Interspersed with numerous Cases.* By CHARLES WILLIAMS JONES. *Second Edition.* By CHARLES MINGAY SYDER, Surgeon. 8vo. pp. 448. S. Highley, London. 1821.

WE know not under what denomination, or in what class, to arrange the [illegible] before us. The first edition was published as the [illegible]duction of a Mr. CHARLES WILLIAMS JONES, of whom the profession knew nothing; but, in the present edition, there is a declaration from Mr. SYDER, intended to convey the fact that every original idea contained in the book is the *bona-fide* property of Sir ASTLEY COOPER, from whom it was derived, during his course of lectures delivered at the united hospitals of Guy and St. Thomas.

That the publication of notes taken under such circumstances, by any of the pupils, without the previous consent, or even knowledge, of the professor, is a practice highly to be deprecated, we have no hesitation in affirming; and we are strongly disposed to believe, that, in the present instance, the knowledge of such a fact, which Mr. Syder wishes us to believe, is likely to injure the reputation of the book, notwithstanding the abundant store of information it may be supposed to contain. After all, we suspect that the whole is nothing more than a mere compilation of the opinions and modes of practice of some of the best operative surgeons of the day, brought before the public in a sort of mysterious dress, with a view to attract that notice which an ordinary book on the principles of Surgery, from the pen of an obscure individual, is not likely, in our days, to obtain. We scarcely need say, that, as a compilation, it is much, very much inferior to Mr. SAMUEL COOPER'S Surgical Dictionary; and that, as an original work, it possesses but very slender claims to our attention.

The subjects treated of in the book are divided into thirty-five lectures. In the first, or introductory lecture, several facts are brought together, relative to the temperature of the blood, its consistency, and property of coagulating, in different constitutions and under different circumstances; it being asserted that, in proportion as the animal is strong, so is coagulation slow; while, in the weak and faint, as we see in patients greatly reduced previous to amputation, the blood coagulates, while on the stump, immediately after the operation.

In considering the strength and frequency of arterial action, the author takes occasion to digress on the effects of stimuli and sedatives. A stimulus is said to increase the momentum of the pulse, while a sedative diminishes it. The effect of *stimuli* depends on the constitution. All stimulants are found to lose their effect by repetition. The utmost stimulating effect produced by opium, is to raise the pulse fifteen strokes in a minute; that of wine, fifty; and this without any adequate sedative effect. There are *sedatives* which have a direct effect on the nerves, without producing a previous stimulus. Belladonna applied to the eye, lead applied to the muscles of the same organ, prussic acid, and digitalis, are examples of this kind. With regard to the effects of heat on the body, they are stated to be universally stimulant.

"The natural temperature of the blood, as I have previously observed, is 98° of Fahrenheit's thermometer, in the human body. A person was put into a vapour-bath, heated to 202°; it raised the pulse to 120. In another, whose pulse was 75, it rose to 164. A third person's pulse, exposed to the same degree of heat, rose only to 145. Dr. Fordyce went into a vapour bath of 120°; his pulse rose to 145: going into a dry heat of 254°, he supported it much better.—The effect of heat on the pulse is to produce a quicker and fuller action: the external veins dilate, and are seen apparently distended, in the warm bath, which always produces perspiration, and is a general stimulus; it has great power in exciting the absorbent vessels, and in weakening the body. In twenty minutes, this power extends to the production of syncope: in five minutes, it only increases the general tone of the vessels."

Heat, when applied to the body with moisture, as in a poultice, relieves by exciting perspiration and unloading the capillary vessels of the part. Fomentations are used with the same view; and, to excite the absorbent vessels, dry heat, by friction or otherwise, will be found beneficial.

The sedative effects of cold are incontestible and curious. It diminishes the frequency of the pulse, if long and slowly applied. When its application is sudden, the pulse is increased. A man, having a pulse of 80, by plunging into the cold bath, it rose to 120; another person, by doing the same, whose pulse was 90, had it raised to 135.

"I have repeated the experiment, that of trying the effect of a partial application of cold, by plunging my arm in snow: my pulse, from beating slowly, became very quick. By the addition of ice to water, lowering it to 33°, my pulse, being 81, was raised to 121; at another time, being 87, to 139. It seems to me to depend on the suddenness of the plunge and the irritability of the person. As this effect of cold is irritating, the pulse, though increased, is a contracted one, and thready. I am not aware that the tonic effects of cold have ever been satisfactorily explained. A debilitated person shall have a quick pulse, but weak, with heat, too, on the skin: the effect of cold is to bring down the pulse to its natural standard, and take off the excessive heat; and it is in this way the cold bath does good in weakly habits, and in typhoid patients."

Cold is applied, occasionally, by means of evaporation. Æther is the best to a warm surface, and has relieved in a few hours, when applied on the temples, severe attacks of head-ach.

"Cold applications are had recourse to for removing inflammation, in some cases, with good effect. The liquor. acet. plumb. dil. c. spir. vin. rec. is an excellent application. It has this double effect, that of cold, and the sedative effect of the lead; and is very effectual in removing inflammation. Few clothes should be put over inflamed limbs. That cold poultices retard the formation of matter, and warm encourage it, must be considered only ideal: heat is so quickly imparted from the body, that, unless it is repeated every ten minutes, nearly the same effect is produced from both."

The lecture concludes with some very good remarks on constitutional irritation; a subject of the highest importance to a surgeon. To relieve this unfavourable state of the system, the author lays down four general rules.

"1st. Take away blood, in proportion to the strength and plethoric disposition of your patient.

"2dly. Restore the secretions of the liver, kidneys, skin, and intestines.

"In children, give calomel and antimonials; for nineteen diseases in twenty, in them, are inflammatory, and this is the best medicine to restore perspiration, &c.; bathe the feet in warm water: by these means you take off the momentum of the blood, and give a healthy action to the secreting organs.

"In adults, it will be better to give calomel at night, and saline purges the following morning.

"3dly. Lessen the nervous irritability by opium, combined with some sudorific.

"And, 4thly. Guide your patient's diet and regimen."

*On Inflammation*, (Lectures 2 and 3.)—When inflammation occurs externally, it is denoted by four marks: redness, pain, swelling, and increased heat. The cause of three of these characteristic symptoms of inflammation, is assigned by the author as being well known; that of the fourth, or increased heat, is said to remain yet a matter of question. It will be recollected that HUNTER denied the occurrence of it altogether, and that he made some experiments to prove the negative position. The author, however, considers those experiments as by no means satisfactory; for there is no doubt of increased heat being found to arise from an inflamed part on the surface of the body. A blister raises a thermometer to 94, while the parts around are only 90.

Inflammation has four terminations:

"1st. Adhesion, from coagulable lymph being thrown out in the cells, and the parts becoming glued together, or incasing the extraneous substance which was the original source of mischief, and all the symptoms existing gradually disappearing. This process, or stage, by some is termed adhesive inflammation, by others resolution, and is the most favourable of each.

"2dly. Suppuration, or a secretion of matter in the centre. From the continuance of the inflammation, the action of the blood-vessels becomes changed, and pus is formed.

"3dly. Ulceration; which eventually, by continued pressure, excites absorption of the surrounding parts, thereby allowing the escape of the extraneous body. This is termed ulcerative or absorbent inflammation.

"4thly. Mortification, or gangrene; in which process the parts, losing their vitality, become dead, and separate from the living, by the suppurative process taking place for their removal."

Inflammation produces different effects in different parts of the body. In the skin, it generally extends a considerable way: it is accompanied by constitutional irritation, shivering, acceleration of pulse, depression of spirits, and prostration of strength.—In the arteries, it is also very extensive; going, sometimes, even to the heart itself: of this an example is adduced, of a man who died after the application of a ligature to an artery, which had produced a violent inflammation.—In the veins, the progress of inflammation is slower.—In the absorbents, inflammation may be distinguished by red lines under the skin, feeling like cords, and frequently terminating at the first absorbent gland.—In inflammation of the membranes, we have not unfrequently serum thrown out, which will occasionally be decomposed, and give rise to the accumulation of gas producing tympanites; as we have ascertained, and demonstrated, in an original essay consigned in another part of the present Number: in this species of inflammation, adhesions very frequently take place, as, for instance, of the pleura to the lungs, of the pericardium to the heart, and of the peritoneum to the intestines.—In the bones, inflammation produces obtuse pain, scarcely to be borne, the constitution suffering more irritation than from the most acute pain.—In the muscles, it produces spasm.—Tendons, when inflamed, slough to a great extent, owing, probably, to the weaker action of the living powers in them.—Nerves are very rarely inflamed; when they are so, the pain is constant, and violently severe.—In the glands, inflammation generally stops their functions: in that of the liver, no bile is secreted.—Inflammation of the viscera affects their different functions: the pulse is generally quick and small; a circumstance which has deterred practitioners from venesection in these complaints, though, without it, there be but little chance of saving the patient's life. The subject of inflammation of the lungs, the heart, and brain, more properly belongs to the consideration of the physician. The author dilates farther on inflammation in the third Lecture, in which he also details, at full length, its treatment.

The fourth Lecture treats of adhesive and suppurative inflammation; in it the author considers the nature of matter and its formation, ulceration, abscesses, hectic fever attending them, union by granulation and cicatrization. Many very useful hints and remarks will be found scattered, here and there, in this lecture, not met with in the generality of surgical publications.

*Ulcers*, (Lecture 5.)—An ulcer is a granulating surface secreting pus: when healthy, the pus secreted is white and thick, and does not adhere to the surface. Every wound, which does not unite by adhesion, must necessarily become an ulcer.

"The only treatment required is, first to apply lint to the surface, which will come off after a few days; and, when removed, granulations will be discovered under it, and a quantity of pus will be also secreted at the same time, and deposited on the surface of the sore. Poultices must then be applied, as they materially assist the rising of the granulations by their gentle stimulus, and are of greater utility than simple dressings of any ointment. In a few days more, they rise as high as the edges of the skin; then lint must be applied, and some unctuous substance, on the edges,—the simpler the better, as that composed only of wax and oil; but be careful the lint does not touch the edges, as it would greatly impede the healing of the parts, by preventing the shooting of granulations to the skin. A formation of a scab, from the evaporation going on, on the surface, will be best prevented by the poultice. This is the treatment required when an ulcer is in an healthy state."

Among the impediments to the healing process, we find enumerated, the luxuriance of the granulations,—their languid condition,—an inflamed state of the parts,—an irritable state of the ulcer,—or a thick and prominent edge of the surrounding skin. When a sore is sloughy and has a black aspect, another impediment is presented to the healing process. Sinuses, or a fungous state of the sore, prevents cicatrization. Another obstacle is the suppression of the menstrual discharge. The author has not mentioned a source of impediment to the healing of sores, which has often baffled the skill and perseverance of the most eminent surgeons,—we mean a diseased state of the surface of the bone in the lower extremities consequent on a gun-shot wound. The ulcer will proceed healthily to a certain point, become contracted, and present the most trifling aperture, which will resist every effort at cicatrization for a number of years. We had occasion to see two singular instances of this kind: the one in a captain of the royal navy; the other in an aid-de-camp of a general officer, at Cambrai, during the occupation of that town by the English troops. In the latter case, the wound was of some years' standing, and reduced to so small a diameter that a common-size probe could with difficulty be introduced. It was caused by a pistol-shot, and was situated over the spinous edge of the tibia. It discharged a small quantity of healthy pus daily, and had healed at different times completely over, but only to break out again. On our return to town, we consulted Sir A. Cooper and Mr. A. C. Hutchison, in behalf of the patient. It was our joint opinion that the diseased state of the bone was an impediment to effective cicatrization, as no foreign body could be suspected to be present, and the ulcer was small and in every respect healthy. With a view therefore to promote a better condition of the bone, injections, with muriatic acid greatly diluted, were suggested. A lapse of three years has since intervened; but the ulcer has not yet healed.

There are some observations on gangrene and mortification in the latter part of this lecture.

Lecture 6 treats of Furunculus, or Boil.

The subject of Wounds is amply discussed in Lectures 7, 8, 9, 10, and 11; the last of these lectures being entirely consecrated to the consideration of gun-shot wounds of the chest, abdomen, and brain; as well as of compound fractures occasioned by gun-shot wounds.

In Lecture 12, the author has taken up the important subject of Poisons, including the virus, which is supposed to produce hydrophobia. One is at a loss to know by what approximation the consideration of morbid effects produced by poisons, taken internally, can be admitted in a purely surgical work. The subject must have been introduced to swell the volume; since in no way can it belong to the province of the surgeon, neither does it lack the assistance of surgeons to be properly treated.

The injuries considered in the succeeding lectures are exclusively surgical. In Lecture 13 we have some good observations on Concussion and Compression of the Brain, Fractures of the Cranium, and their treatment; and the Formation of Matter within the Skull.

"The most certain mark of the formation of matter going on, is the patient being seized with violent shiverings, which often remit, similar to those of an intermittent fever. The sooner relief is had recourse to, the more likely is it to be attended with success. The seat of the matter is similar to what takes place in extravasation,—namely, in three places: first, between the dura mater and the skull; secondly, on the pia mater; and, lastly, in the brain itself. When situated on the dura mater, the only and quickest relief is by means of the trephine: the matter escapes, after a little while, through the bone. The perforator alone is occasionally used with success. This latter instrument is proper when the skull is very thin, as in very old or infantine subjects: here the saw would be likely to wound the dura mater, which must always be cautiously avoided. I wish I could say that the operation for the discharge of matter, when seated between the dura mater and the skull, was always attended with success, or even in the majority of instances; but I am sorry to state, from what I have seen, that I believe there are but few that survive: and there is still less chance when matter is on the pia mater, much less in the brain itself; for, if an opening be made through the dura mater, the probability is that the patient will die from inflammation."

We have had occasion to perform the operation of trephining, on-board H. M. ship Swiftsure, on a petty officer, afflicted with caries of the parietal bones from syphilitic action, where large portions of the diseased bones were removed at two distinct periods, so as to bring the dura mater into view, and thus give a free exit to the pus collected on its surface. The patient recovered.*

The operation of trephining is next described by our author.

Lecture 14 is on Hydrocele. The palliative, as well as the radical, treatment of this complaint is mentioned; and the cure by injection fully detailed. The author thinks that, in using Sir James Earle's injection, equal parts of wine and water are not too strong; or one part of brandy to five of water may be employed. Simple as the operation of dividing the integument, and throwing the injection into the tunica vaginalis, may appear, the lecturer properly observes, that it is not always free from danger. In support of this assertion, he brings forward a case where the operation proved fatal, from being *incorrectly* performed. One cannot help shuddering at this wanton waste of human life.

"An old man had the operation performed on him in Guy's Hospital. About two years after, he was again admitted, and it was done by a dresser, and in a proper manner, as he concluded; but, during the operation, the patient appeared in excruciating pain; to relieve which, the dresser removed the elastic bottle, but was much surprised not to see any fluid return with it: neither was he able, even by pressure, to get out any of the injection, it having passed into the cellular mem-

* See a paper entitled *Caries Ossium Cranii ex Syphilitide*, in the 29th volume of the London Medical and Physical Journal; by A. B. Granville, m.d. Surgeon R.N.

brane, ***owing to the canula not properly fitting the trocar***, and, the more he pressed, the more the injection passed towards the abdomen. The consequence was excessive inflammation, succeeded by mortification, which carried him off."

The two succeeding lectures are on Aneurism, and the Operations for the removal of Diseases of the Eye. Lecture 18 treats of Ischuria and Puncturing the Bladder. The subject of Calculi, their situation, symptoms, chemical composition, cause of formation, and medical treatment, is fully entered into in the next lecture. The old and new mode of performing the operation of lithotomy are likewise detailed. Two great improvements in this particular branch of surgery are here wholly omitted, one of which is due to Sir Astley Cooper himself, the substance of whose lectures our author pretends to have condensed in the present volume. This omission would induce us to suspect the title of this work to have been assumed as a *nom de guerre*,—as a passport to public patronage and approbation. Else how came Mr. Jones, alias Mr. Syder, to neglect detailing the mode of extracting small calculi from the bladder through the urethra, by means of the long peculiar instrument, which Sir Astley Cooper had most ingeniously devised, long before the date of Mr. Syder's introductory address to his volume. The second omission is pardonable, because it relates to SANSON's new operation, improved by VACCA, a full detail of which was only published within the last few months; although the report of BARBANTINI having successfully extracted calculi from the bladder by the rectum, was made known in England as early as January 1821, six months previous to Mr. Syder's publication.

The Diseases of the Testicles and of the Breasts occupy the 20th and 21st Lectures. Amputation is treated of in Lecture the 22d; after which, we have a very ample history of Hernia, in all its modifications, stages, and situations, taking up four whole lectures.

Dislocations, Fractures, Syphilis, Scrofula, and Tumors, form the subjects of the concluding portion of the volume; respecting which we have said enough to show that it is, by no means, a superficial or a useless performance, though not at all wanted in the present glutted state of the market in regard to elementary works of surgery.

### DIVISION II.

### FOREIGN.

### Art. III.—BOTANY OF THE UNITED STATES.

A. *American Medical Botany; being a Collection of the native Medicinal Plants of the United States, containing their botanical history and chemical analysis, and properties and uses in medicine, diet, and the arts, with coloured Engravings.* By Jacob Bigelow, M. D. Rumford Professor and Lecturer on Materia Medica and Botany in Harward University. Boston, Cummings and Hilliard, 1817—1821. 6 Nos. or 3 Vols. imp. 8vo.

B. *Vegetable Materia Medica of the United States, or Medical Botany; containing a botanical, general, and medical history of medicinal plants indigenous to the United States; illustrated by coloured Engravings, made after original drawings from Nature.* By W. P. C. Barton, M. D. Professor of Botany in the University of Pennsylvania. Philadelphia, vol. 2. 4to. 1817—19.

A large portion of the first of these two works has been before the public long enough to have been justly appreciated. Of the second we had occasion to speak favorably when the first edition made its appearance.

That botanical science is flourishing among the Americans there can be no doubt. To the three illustrious names they already possessed as botanists, Clayton, Bartram, and Colden, they may now add those of Mecklenberg, Elliott, and Nuttall, as well as those of the authors we have selected for review, Barton and Bigelow, who have successfully cultivated the study of botany, particularly as it applies to the purposes of medicine.

It was, indeed, to be expected; from a people who lack neither means nor inclination, that, as soon as the more weighty considerations of consolidating a nascent commonwealth began to give way to the more pleasing reflections on the territorial beauties of their country, they would direct their attention to some of the productions of nature, and particularly to those of the vegetable kingdom, which Providence seems to have scattered around them with a bountiful hand. We believe that hardly a single vegetable of North America has been described, which cannot be indicated as growing, spontaneously, within the present enlarged limits of the United States; and, it is fair to presume, that the botanical collections, floras, and other works on botany which have appeared under the comprehensive title of Descriptions of North American Plants, contain, scarcely, a single specimen which cannot be, rightly, appropriated to the states and territories of the American Republic, stretching, as they now do, from the Lakes to the Gulf of Mexico in one direction, and, in the other, from the Atlantic to the Pacific

Ocean,—embracing, in these boundaries, every conceivable variety of situation, climate, and soil.

Having premised these considerations, it will not be improper to take a brief review of the principal botanical works that have been published relative to the United States; and next to inquire how far these works will go towards affording us the materials for a perfect history of the plants of the United States.

In prosecuting this inquiry, we shall draw largely on the ample store of information contained in the last number of the North American Review, from whose pages we shall take leave to give occasional quotations.

There are only vague and cursory notices of the vegetables of the United States given by early travellers previous to the 17th century. GOSNOLD, the first who explored New England, the least favored, perhaps, in its vegetable aspect, of any part of the United States, represents himself to have been utterly astonished at the rich appearance of the country. Observations, like these, however, are not of much use to the modern botanist, excepting that they sometimes enable him to establish the birth-place of plants which are now common to both worlds. Thus, for instance, it is known that some of the most common plants in the United States, such as the Chrysanthemum leucanthemum, were introduced from abroad; and that others, like the Pyrola umbellata, which has been used as a medicine among the aboriginal inhabitants of the country, are indigenous to both Europe and America.

CORNUTI is the earliest writer of a systematic work on the botany of the United States. It is entitled a History of the Plants of Canada, and was printed at Paris in 1635, in a small quarto volume in Latin, with coarse engravings of sixty or seventy of the most remarkable plants found in New France. Some information with regard to the botany of the United States was also given in several other works published in the same century, the most complete of them being the natural history of North Carolina by BRICKELL, printed in 1737.

It was about this same period that CATESBY's Natural History of Carolina appeared in numbers, with splendid coloured plates, etched by the author, who, having previously spent seven years in Virginia, returned to America at the suggestion of his patron Dr. Sherrard, and with the assistance of the Earl of Macclesfield and Sir Hans Sloane.

The next work on the plants of the United States, or that part of America which is now under that denomination, is of less pretension than the one just mentioned, but of far more value as a scientific performance; namely, the Flora Virginica, the joint production of Clayton and Gronovius, assisted by Linnæus himself. John Clayton emigrated from England to America, and during a long life of eighty-eight years he assiduously cultivated the science of botany in which it is well known that he attained a high rank among the learned of Europe. Clayton's herbarium was purchased by Sir Joseph Banks, the late illustrious patron of science. The Flora Virginica is the oldest accurate systematic work on American Botany; the Linnæan classification, then but little known in Europe, having been adopted in its compilation. A new edition of the Flora was some years ago undertaken by the ancestor of one of the authors before us, but circumstances and occupations of a different sort prevented his accomplishing the task he had imposed on himself.

Bartram, a native of Pennsylvania, esteemed by Linnæus the best practical botanist of the age in which he lived, communicated to the different academical societies of Europe the fruit of his inquiries; and, in a paper published among the Acta Societatis Upsaliensis, there is an enumeration of several hundred American plants drawn up by CADWALLADER COLDEN, the able historian of the Five Nations. Linnæus had a fourth American correspondent, in the person of Dr. ADAM KUHN, who became the first public teacher of botany in the American States.

"The first fruits of the peace, however, were a native production, namely, an Account of the Vegetables indigenous to this Part of America, published in 1785 in the Memoirs of the American Academy, by the venerable Dr. Cutler. Considering the time when this paper was drawn up, and the little attention which had then been paid to botany in New England, it was certainly, notwithstanding its want of completeness and accuracy, a meritorious production, from its bringing into notice several valuable plants, whose properties were very imperfectly understood previously to its appearance."

The Arbustum Americanum of H. MARSHALL was published in 1785 at Philadelphia. This work, containing an account of the American forest trees, was held in considerable estimation, and was translated into French.

"Next after these appeared the Flora Caroliniana. Of its author, Thomas Walter, we have never happened to meet with any notice in print; but in his preface he informs us that all his specimens were collected within an area of two hundred miles in circumference, which, from the date of the preface, would seem to have been on the banks of the river Santee in South Carolina. His work was printed in London in 1788, in Latin, containing the essential characters and specific descriptions of more than a thousand plants, arranged according to the principles of the sexual system. It is valuable as containing a large number of species, and nearly thirty genera, then for the first time described; but as it merely gives the botanical differences, without any allusion to the habit, soil, or other circumstances connected with the plants, it is now superseded by later publications; although few authorities are more frequently quoted, or more relied upon, than the name of Walter."

It should be mentioned, also, that a gentleman of Milan, named Luigi Castiglioni, who spent nearly three years in the United States between 1785 and 88, published some remarks on the most useful vegetables of that country in his travels, printed after his return to Italy.

We are now arrived at the epoch when the two Michaux, senior and junior, were dispatched from France to America to pursue botanical inquiries, which led to the publication of two of the most sumptuous works on forest trees and American oaks, that have ever appeared in any country.

"Michaux collected the materials for his work in the course of two voyages to America, concerning the first of which he published a volume, which contains some valuable hints spread out over a considerable surface. It is the plan of his History of our Forest Trees to unite the advantages of a work strictly botanical and of one relating to the useful arts, but especially to collect all the scattered details, which books or experience could furnish him, with respect to the application of the various kinds of wood to the purposes of life. Botanical descriptions can easily be made or found; but, in order to ascertain their useful properties, it was necessary to consult artisans in almost every branch of practical mechanics, to frequent the dock-yards or work-shops, in which wood was employed, and in short to gather information from every attainable source. From these inquiries Michaux has obtained a most extensive collection of curious and important facts, which, although rather belonging to the application of botany than to botany itself, are nevertheless essential to the complete knowledge of the plants of the United States. For, besides the commercial and practical uses of our trees, we have a very perfect account of the inflorescence, fructification, growth, and botanical habit of them individually considered, as also many interesting facts with regard to them taken together as composing forests. With respect to the nomenclature of this work it may be observed, that Michaux has been charged with making needless innovations in long established names, sometimes without any intimation of the change. But the great accuracy of its beautiful figures, at the same time that it serves to correct any misunderstanding that might arise from occasional confusion in the specific names, gives this History of our Forest Trees a permanent value in technical botany."

Mecklenberg and Dr. Barton are the two next American botanists, of whom every one has agreed to speak in the highest terms of gratitude. Both were natives of Lancaster, and the latter had filled the botanical chair in the university of Pennsylvania, in which capacity he died in 1815, leaving behind him two ingenious and useful works, the one entitled an Essay towards the Materia Medica of the United States, the other Elements of Botany.

"In the light of an elementary treatise, the last work must yield to many others, to Smith, to Sprengel, and to De Candolle. Still it derives a peculiar attraction, in the view of Americans, from the circumstance, that it brings our own plants into notice by frequent reference to them, and by means of its coloured figures, which Barton was very solicitous to have faithfully executed. In all the scientific writings of this author, however, a diffuse inelegant style, and an immethodical arrangement, into which the hurry of his professional pursuits betrayed him, are too remarkable to pass unnoticed, especially as he published almost nothing but incomplete tracts, whose faultiness their very title of *fragments, collections*, and the like, sufficiently indicates. We apprehend, therefore, his botanical fame is built most surely on the gratitude of those naturalists, who were aided by his liberality in making the researches, which his public duties withheld him from undertaking in person."

Mecklenberg died in the same year whilst occupied in preparing for the press a second edition of his Catalogue of the Plants of North America. His herbarium was purchased after his death by a number of gentlemen, and presented to the American Philosophical Society.

Among the subsequent writers on American botany, we cannot omit mentioning the name of PURSH, a Muscovite, who travelled through different parts of the United States, where he procured so many plants, that, in 1811, he came over to England with one of the most perfect collections that ever crossed the Atlantic. Pursh was countenanced by Sir James Smith in this country, by Sir Joseph Banks, M. Lambert, and the historian of Lorenzo. In 1814 he published his Flora Americæ Septentrionalis; and, having afterwards returned to America, for the purpose of exploring the botany of Canada, we are told in Silliman's tour, that he died between Hartford and Quebec, in the year 1820, not having, yet, completed his collections towards a Canadian flora.

The order of time now requires us to advert to Mr. ELLIOTT's Sketch of the Botany of South Carolina and Georgia. This work promises an account of the plants of a most interesting part of America, and it is to be regretted that the author's private occupations and station in life have hitherto prevented him from bringing it to a conclusion.

To those who possess and wish to study American botany in Pursh's Flora, Mr. Nuttall's valuable work on the genera of North American plants published in 1818, becomes an indispensable supplement.

"This work abounds with extensive remarks, the result of long personal observation in different sections of the Union, especially in the Southern and Western States, in which last the author had a most ample field of research and discovery. His principal design was to describe the genera of our plants and merely enumerate the species. But he does much more than he promises, not only introducing a large number of new species detected by himself, but continually enriching his list of the old species with instructive remarks on their habit or character, and often going so far as to give us a full account of all the species of a genus. Among the genera so treated we may notice Viola, Rhexia, Œnothera, Laurus, Lobelia, Polygala, Carduus, Erigeron, Inula, Aster, and Solidago, as particularly rich in interesting botanical suggestions, from which we have repeatedly derived great advantage. But the author has been most elaborate and masterly in his delineation of the essential characters of the genera, which it is the primary object of his work to elucidate. He has proposed above sixty new genera, in several cases constructed by means of plants before imperfectly known, but chiefly by the subdivision of old genera."

Soon after the publication of Nuttall's genera, appeared Eaton's Manual of Botany for the Northern and Middle States, which has since been reprinted.

It only remains for us to speak of two contemporary botanists, the authors now before us, Dr. William P. C. Barton, a nephew of the eminent botanist of the same name, and Dr. Bigelow. The former has published books on the subject of American botany, of greater pretensions, than almost any other living author. In 1815, he printed a Prodromus of his intended Flora Philadelphica; in 1817, he began the publication of his Vegetable Materia Medica of the United States, the completion of which we announce, having on a former occasion mentioned the appearance of the first volume only. This work, the title of which stands second at the head of the present article, gives an account of forty-eight plants, with coloured engravings; including, first, the botanical characters of the genus and species, adopted from the best authors, with a note of synonymes, and copious references to writers who treat of the plant; next, a general botanical history of each plant, executed in a cursory manner; and, lastly, its chemical analysis, medicinal properties, and economical uses.

The work manifests considerable industry in the compilation of facts and statements from various books; but it is rather unfortunate to find that, in the parts relating to medicine, it appears to be almost entirely a compilation. Dr. Barton appears rarely to have performed any experiments on the chemical organization of plants he describes, and seldom to have ascertained their reputed effects on the human system by personal investigation; so that, in his remarks on the medicinal properties of plants, he frequently discovers his little acquaintance with the subject. The figures were drawn, and partly coloured, by the author; many of them correctly enough, but almost all of them betraying marks of great haste and inattention. Previously to the completion of this work, Dr. Barton gave the public, in 1818, his Compendium Floræ Philadelphicæ; and he is, at this moment, issuing an expensive Flora of North America.

Should he persist in bringing forward his Flora Philadelphica, we trust he will either mend his Latin, or employ a more able hand to write the descriptions, so that they shall not exhibit the specimens he has given us in the present work, indubitably exhibiting glaring marks of disregard of the most common rules of grammar and concord. How would an Eton boy grin at such concordances:—"*Baccæ* semina tria *complectens.*" "Foliola *subæquales*," instead of *subæqualia.* "Flores *magnes!*" (which, by the bye, the author, in his errata, corrects "*magnæ*,") cum multis aliis.

Dr. Bigelow is a man of very different stamp, and his productions do real credit to the country to which he belongs. His edition of SMITH's Introduction to Botany prepared the public to receive with favour the Florula Bostoniensis, which came out a few months after the Introduction; and both, together, were precisely of that popular description which was best adapted to advance the name of the author, and, with it, the interests of science.

Dr. Bigelow has since published several tracts connected with botany, to which we merely allude, that we may come the sooner to the consideration of his American Medical Botany, now lying before us.

The contents of this work are a detailed account of sixty of those indigenous or naturalized plants, which are best capable of application to medical use, or seem otherwise remarkable on account of their ascertained or reputed effects upon the human body. It not having been our intention to enter into an examination of any thing more than the botanical part of the work, we shall only observe, as to the rest, that neither Schœpf nor the Bartons materially increased the sum of our information concerning the chemical or medicinal properties of the vegetables of the United States. Schœpf pretends to do nothing more than record, with the most implicit credulity, whatever, to translate his own words, he could learn "from physicians, empirics, peasants, and old women;" the natural consequence of which is, that his book is full of stale drugs of exploded virtue, almost every one of which he represents as a panacea. The Bartons present us with very few, and those but partial, investigations of the chemical constitutions of plants, and still fewer satisfactory proofs of their practical utility. Dr. Bigelow, on the contrary, has made careful experiments on the analytical structure of the plants which he selected for examination:

many vegetables, of supposed efficacy, he has proved to be feeble and inert; others, before of doubtful utility, he has established among the happiest agents in the materia medica; and none has he left without making some perceptible addition to the stock of science. Passing from these considerations, we find that he has not only described the botanical properties of his plants with great accuracy, which is more than can be said of his predecessors, but has also done it with perspicuity and elegance of expression. Every article abounds with important botanical observations: often, too, the character of a plant leads to instructive remarks connected with it, like those upon generic distinctions in Gillenia trifoliata, upon the communication of plants between Europe and America in Ranunculus bulbosus, upon the scenery of our forests in Rhododendron maximum, —all of which are the more grateful for being unostentatiously introduced. Eight of the plants in the Medical Botany are also described and figured in the younger MICHAUX, namely, Laurus sassafras, Magnolia glauca, Cornus florida, Juglans cinerea, Kalmia latifolia, Liriodendron tulipifera, Rhododendron maximum, and Juniperus Virginiana: but a comparison of the respective articles in each book will render it obvious that Dr. Bigelow is not a copyist; for, in these very articles, the Medical Botany abounds with interesting facts, which it did not enter into the design of Michaux to notice, if they ever came to his knowledge. The same comparison, we think, will end in a conviction of the greater accuracy of the figures in the Medical Botany; inferior, as they certainly are, to those of Michaux in brilliancy of colouring, and in that graceful, lively, finished representation of verdant nature, which evinces the perfection of modern art in Europe. Thus, if we compare the Liriodendron tulipifera in each, we perceive a more vivid green in the print of Michaux than we do in that of Bigelow: but the latter is drawn from the best-chosen specimen of the two; and the flower in Michaux is spread open so far as to conceal the calyx, and give an artificial appearance to the petals and stamens; both which defects are avoided in the plant as delineated in the Medical Botany. Bigelow's work comprises most of the plants figured in Barton's Materia Medica, and also many beside; and, although the prints of the latter derive a superior freshness from the circumstance that the colours were put on by the hand, whilst the former came coloured from the plates, still the greater finish and fidelity of the figures in the Medical Botany entitle them to higher regard than those of the Vegetable Materia Medica. Whether we regard Dr. Bigelow's work as a specimen of art, or as a scientific exposition of facts, we think it may be safely put into the hands of foreigners, together with CLEAVELAND's Mineralogy, in proof that the Americans no longer mean to learn from them how to prize the riches contained in their forests and on their mountains.

Here terminates the literary history of American botany in the United States. That much has been done to promote this branch of natural science in that country, we have brought forward sufficient evidence to prove. But the details of that very evidence also render manifest, how defective are still their collections of indigenous plants; and how numerous are the deficiencies to be made up by native Americans, to complete the history of the vegetable riches profusely scattered over the extended surface of their territory.

---

