# Errata in Last Number

**Published:** 1822-04

**Authors:** 


ERRATA *in last Number.*

Page 235, line 20 from top, cancel the epithet *long.*

